# Artificial intelligence in breast imaging

**DOI:** 10.1590/0100-3984.2023.56.5e1-en

**Published:** 2023

**Authors:** Juliana Mariano R. B. Mello

**Affiliations:** 1 Hospital de Clínicas de Porto Alegre (HCPA), Porto Alegre, RS, Brazil. julianamrbm@gmail.com / jmrmello@hcpa.edu.br.

Much has been discussed regarding artificial intelligence (AI) and its practical implications for the daily workflow of radiologists^(^1^-^3^)^. While there are ongoing debates regarding legal and data protections, AI is gaining ground, especially in the scientific community, where everything usually starts and where ideas about future directions in the medical field are initially influenced. There have been a number of studies comparing the diagnostic performance of AI with that of radiologists in various countries. In a recent meta-analysis, AI demonstrated diagnostic accuracy equal to or greater than that of radiologists^(^1^)^. However, the authors noted that, in comparison with radiologists, AI methods had higher sensitivity but lower specificity, suggesting a potential practical use of AI in breast cancer screening, assuming that a subsequent detailed analysis will be performed by a radiologist. It is noteworthy that the authors of that meta-analysis cited potential conflicts of interest, detailing their relationships with AI software manufacturers^(^1^)^.

Bringing national AI research data to the forefront, this issue of Radiologia Brasileira presents an interesting original article reporting data collected at a referral center for breast imaging in Brazil^(^4^)^. Among the main contributions of the article is the finding that the use of AI in conjunction with analysis by a radiologist has a positive impact, reducing the rate of false negatives in the ultrasound assessment of breast nodules. The article also underscores the need to determine whether the clinical findings correlate with the imaging findings, as assessed solely by the radiologist, who also takes into consideration the medical history of the patient and individual family risk factors. As emphasized in the final recommendations in the article, the radiologist evaluation is essential because it takes into account factors beyond the image analysis. The text emphasizes the importance of comparing the current examination with previous imaging examinations of the same patient. However, the study has some limitations, such as its retrospective nature and the fact that it was a single-center study. In addition, the authors selected specific cases that had previously been evaluated by radiologists, potentially introducing a selection bias by not including all examinations previously evaluated by radiologists at the center. Nevertheless, it is a rich study with a significant sample size, considering unique aspects of the reality in Brazil. It should be borne in mind that, as mentioned by the authors, breast ultrasound at major referral centers in Brazil is performed by specialist physicians, rather than by technicians or technologists, as is common at many centers around the world, even in developed countries^(^5^)^. This difference in the execution of the examination is crucial for achieving satisfactory results.

Certainly, the best scenario for breast ultrasound accuracy could be identified in future prospective studies, which might demonstrate the positive impact that the use of AI tools in conjunction with radiologist analyses has on diagnostic precision in breast ultrasound reports. In addition, with the increase in medical malpractice lawsuits in Brazil and worldwide, the use of AI may provide greater medico-legal security for the physicians involved and for the institution at which a patient was treated. The use of AI can also have a positive impact by reducing the costs associated with false positives in breast ultrasound examinations, which are operator-dependent. However, the size of that cost reduction will depend on the operational costs of incorporating AI into the daily practice of radiologists.

There is still a need for multicenter prospective studies, preferably studies that are interventional rather than merely observational^(^6^)^. Such studies should encompass various diagnostic scenarios, in the public and private sectors, to demonstrate greater confidence in the use of AI in combination with radiologist evaluation. Although we do not possess magical powers of prediction, it is evident that AI is gaining strength in the international radiology context^(^7^)^. However, determining the economic impact of AI implementation on radiology as a whole will require a more detailed analysis, including aspects such as the operational costs of acquiring and maintaining software in practice^(^8^)^. If an increase in the number of breast cancer screenings using digital mammography in conjunction with AI is a real possibility, it could lead to advances in early diagnosis for many patients. Radiologists could use AI as an ancillary tool to increase the overall efficiency of their work.
